# Supplementary material for: Integrative network analysis reveals molecular mechanisms of blood pressure regulation
Source: Mol Syst Biol. 2015 Apr 16;11(4):799. doi: 10.15252/msb.20145399 (PMC4422556; doi:10.15252/msb.20145399)

Supplementary Fig S1: The distribution of SBP and DBP in the 3679 FHS participates who were not receiving anti-hypertensive treatment. A) Histogram of SBP; B) Histogram of DBP.

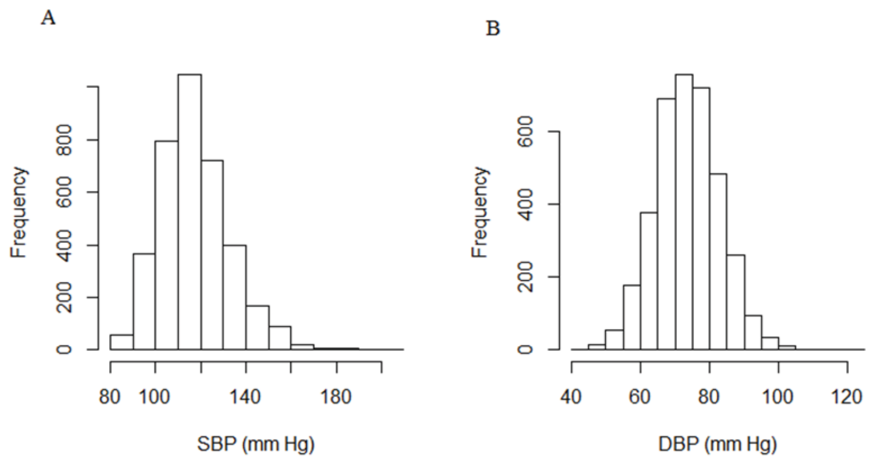

Supplement: Supplementary file 1 — Supplementary Figure S1 [file MSB-11-799-s001.pdf]
