# Supplementary material for: Integrative network analysis reveals molecular mechanisms of blood pressure regulation
Source: Mol Syst Biol. 2015 Apr 16;11(4):799. doi: 10.15252/msb.20145399 (PMC4422556; doi:10.15252/msb.20145399)

Supplementary Fig S2: Dynamic tree-cut plots of the coexperssion networks using gene expression data adjusted and unadjusted for cell counts, respectively. A) network from cell count adjusted analysis; B) network from cell count unadjusted analysis. The gene clustering tree (dendrogram) was obtained from an average linkage hierarchical clustering of adjacency-based dissimilarity metric. The dynamic tree-cut algorithm was then used to choose the cut-off for module identification. The modules are highlighted as color blocks. The height of the tree (dendrogram) indicates the dissimilarity score among genes.

A

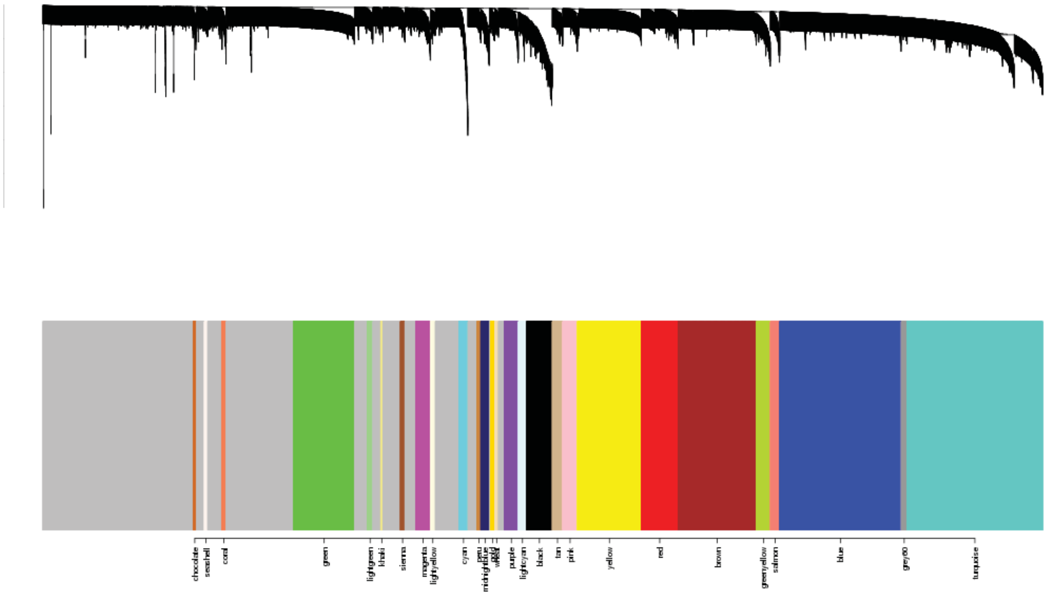

B

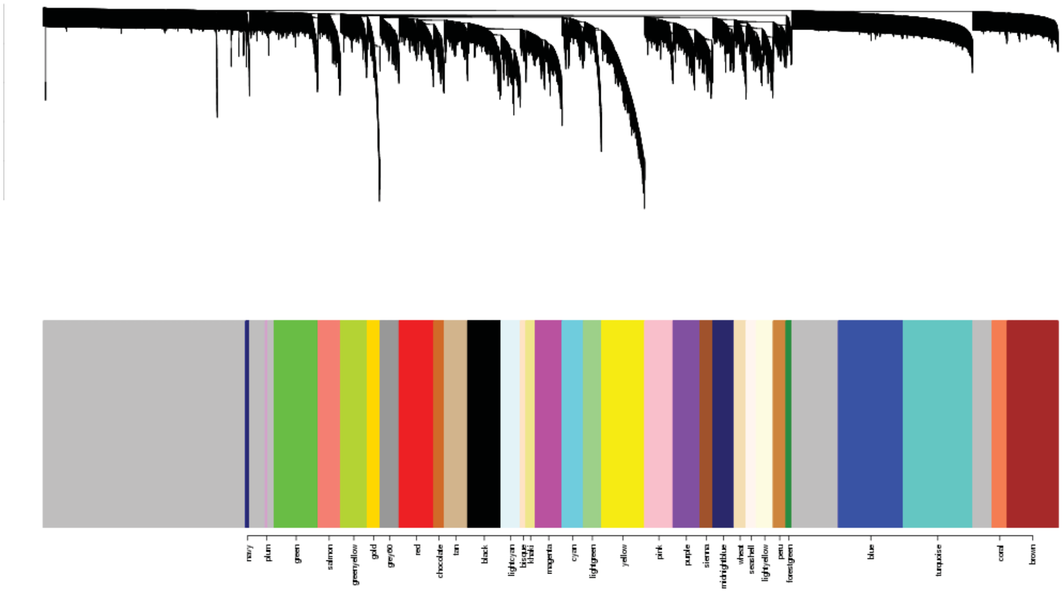

Supplement: Supplementary file 2 — Supplementary Figure S2 [file MSB-11-799-s002.pdf]
