# Supplementary material for: Integrative network analysis reveals molecular mechanisms of blood pressure regulation
Source: Mol Syst Biol. 2015 Apr 16;11(4):799. doi: 10.15252/msb.20145399 (PMC4422556; doi:10.15252/msb.20145399)

Supplementary Figure S3. Co-expression modules and their correlation with BP. A) The correlation of eigengenes of each BP coexpression module (coEM) with SBP; B) The correlation of eigengenes of each BP coEM with DBP; C) The enrichment of BP top signature genes in each BP coEM. The y-axis is the –log10 transformed p value.

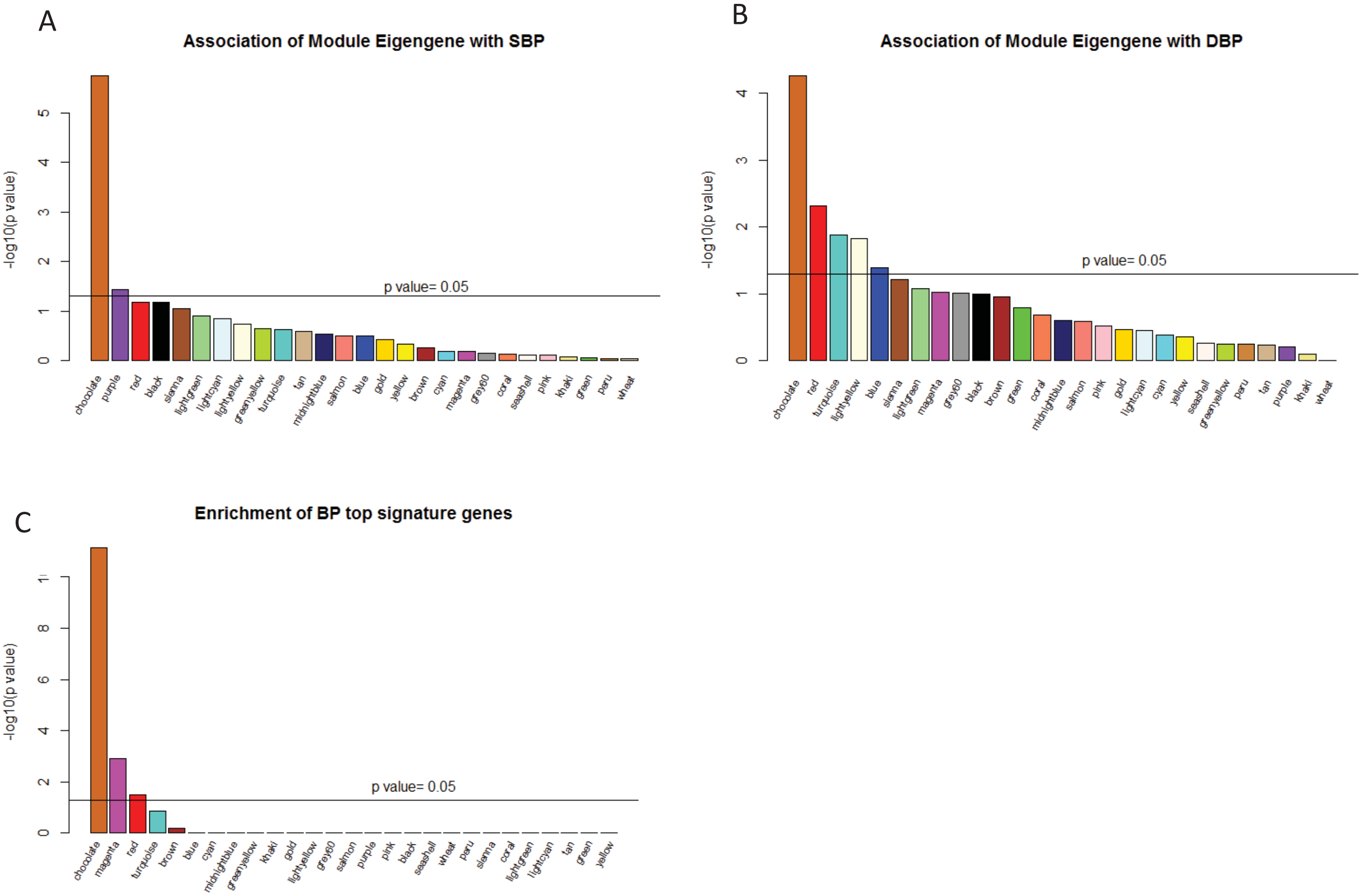

Supplement: Supplementary file 3 — Supplementary Figure S3 [file MSB-11-799-s013.pdf]
