# Supplementary material for: Integrative network analysis reveals molecular mechanisms of blood pressure regulation
Source: Mol Syst Biol. 2015 Apr 16;11(4):799. doi: 10.15252/msb.20145399 (PMC4422556; doi:10.15252/msb.20145399)

Supplementary Fig S4: Coexpression module comparison between the cell count adjusted and unadjusted analyses. Modules from the adjusted analysis are listed along the Y axis and modules from unadjusted analysis are along the X axis. The numbers of genes in each module are listed after the module name. The number in each cell of the matrix indicates the number of genes that overlap between the two modules at the intersection. Color strength indicated the –log10 transformed overlap p values assessed by Fisher's exact test.

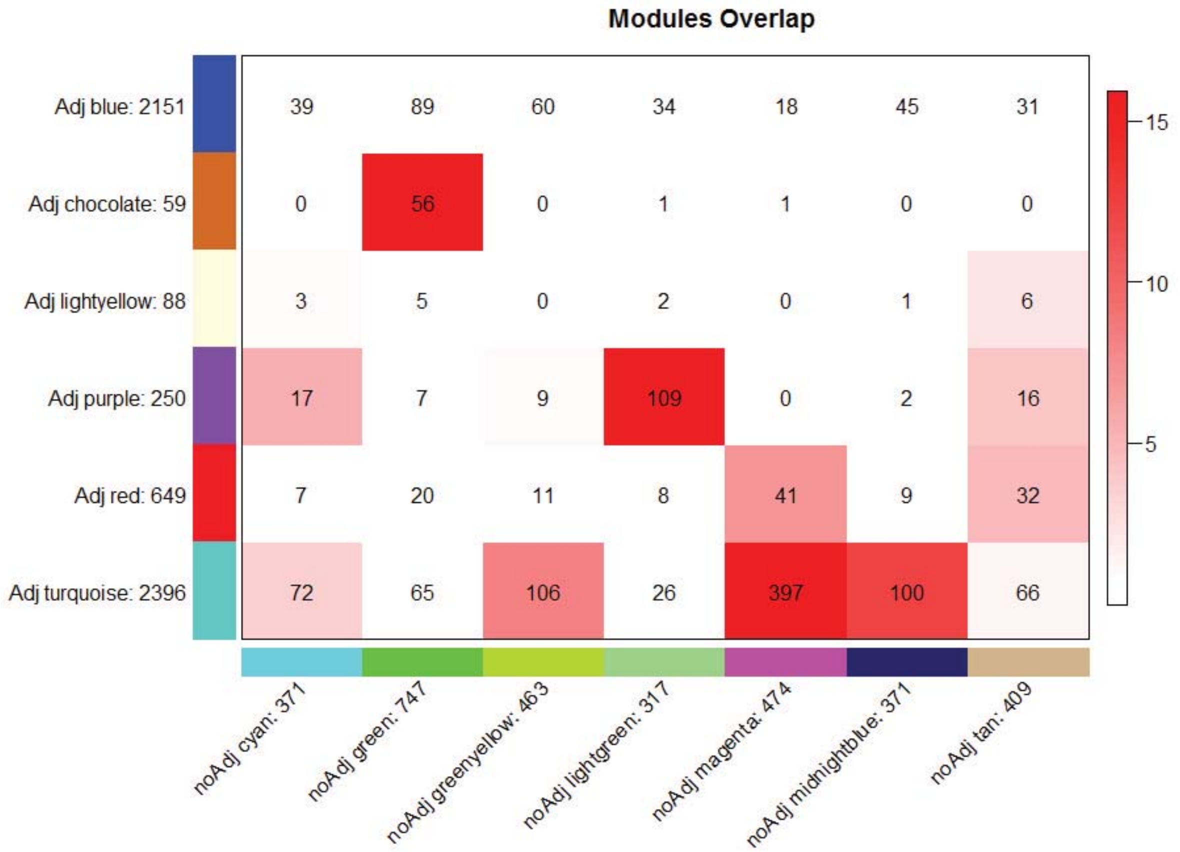

Supplement: Supplementary file 4 — Supplementary Figure S4 [file MSB-11-799-s014.pdf]
