# Supplementary material for: Integrative network analysis reveals molecular mechanisms of blood pressure regulation
Source: Mol Syst Biol. 2015 Apr 16;11(4):799. doi: 10.15252/msb.20145399 (PMC4422556; doi:10.15252/msb.20145399)
Supplement: Supplementary file 5 — Supplementary Figure S5 [file MSB-11-799-s003.pdf]

Supplementary Fig S5: Example subnetworks derived from three top KDs. A) *WNK1* derived subnetwork; B) *BHLHE40* derived subnetwork; C) *NRC2C2* derived subnetwork. Green node indicated BP correlated genes; and Turquoise node indicated genes in the BP putatively causal coEMs.

A

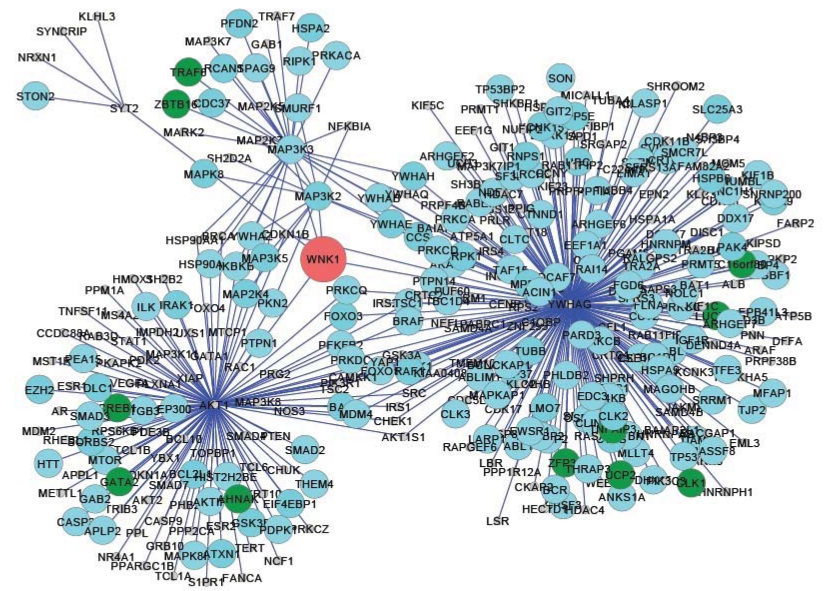

B

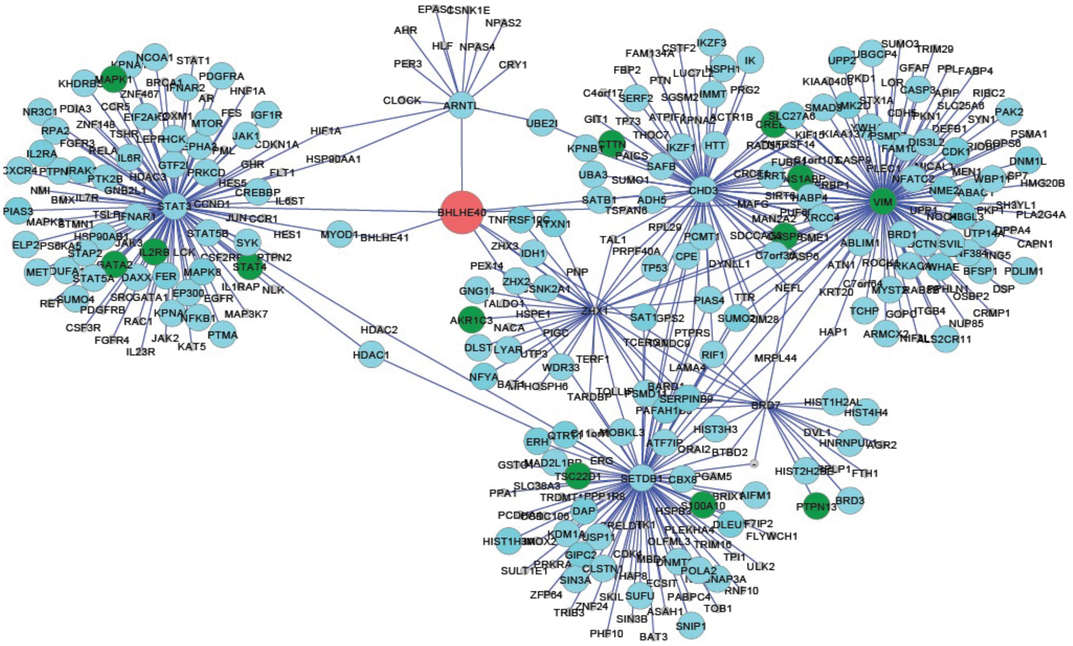

C

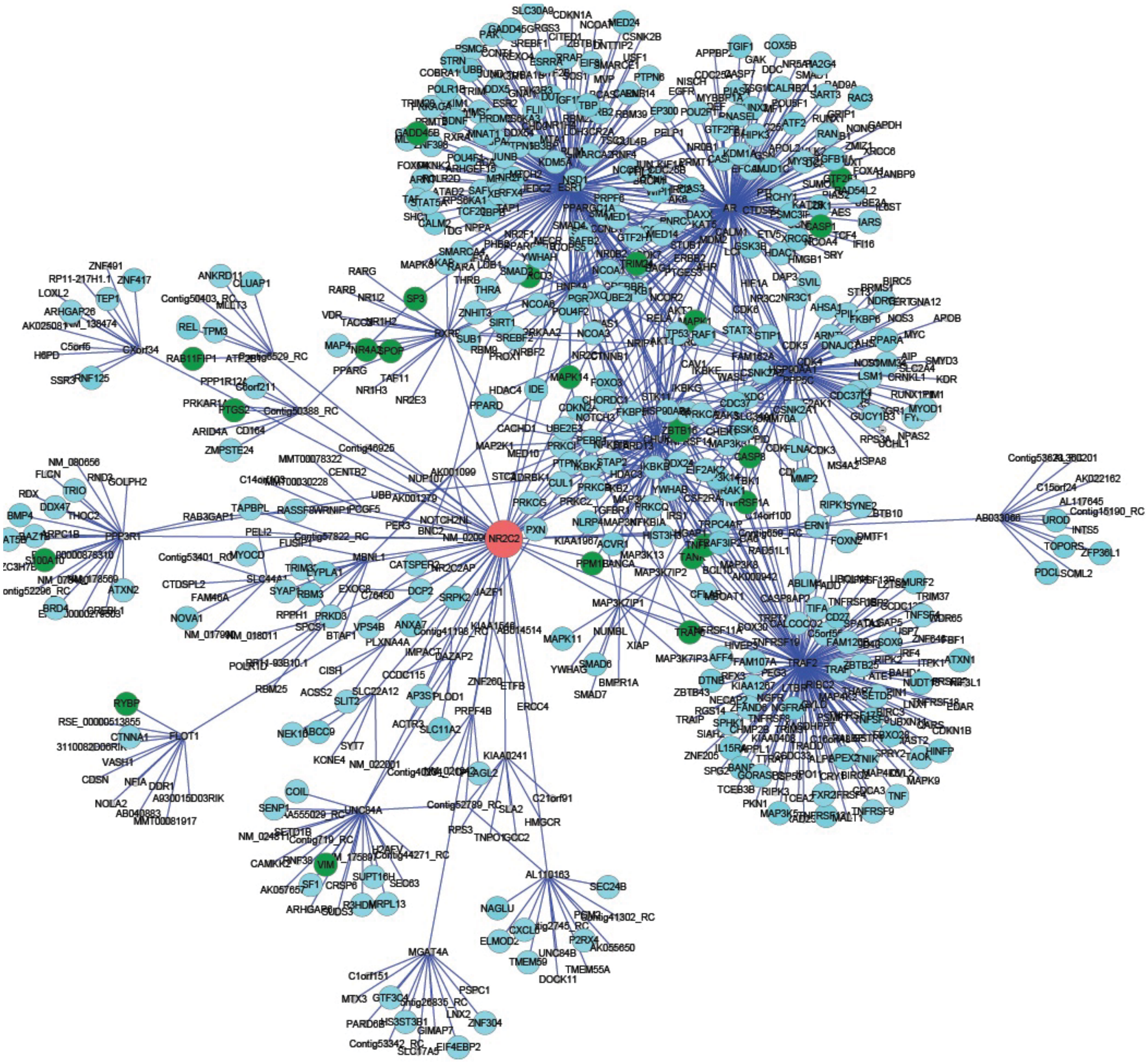

NR2C2
AR
ESR1
TRAF2
RXRB
PPP3R1
MBNL1
UNC84A
FLOT1
MGAT4A
AL110163
AB033060
CXorf34
MAP3K7IP1
MAP3K7IP2
KIAA0241
PPARG
PPARD
HDAC4
IDE
MAPK14
MAPK11
SMAD6
SMAD7
BMPR1A
XIAP
NUMBL
PXN
PRKCG
CUL1
NLRP4
PPM1B
RYBP
VIM
SP3
NR4A3
SPOP
PTGS2
RAB11FIP1
REL
TPM3
ANKRD11
CLUAP1
TEP1
ZNF417
ZNF491
LOXL2
SSR3
RNF125
H6PD
TRIO
GOLPH2
BMP4
DDX47
THOC2
ATXN2
BRD4
NOVA1
SPCS1
BTAF1
VPS4B
PLXNA4A
ANXA7
DCP2
SRPK2
JAZF1
CATSPER2
DAZAP2
CCDC115
PLOD1
SLC11A2
PRPF4B
ETFB
ERCC4
ZNF260
SLIT2
NEK10
ABCC9
KCNE4
SYT7
CISH
ACSS2
COIL
SENP1
CAMKK2
SF1
MRPL13
SEC63
SUPT16H
NAGLU
ELMOD2
TMEM59
UNC84B
DOCK11
TMEM55A
P2RX4
SEC24B
AK055650
GIMAP7
LNX2
ZNF304
PSPC1
MTX3
PARD6B
EIF4EBP2
C1orf151
GTF3C4
HMGCR
C21orf91
SLA2
RPS3
ERN1
FOXN2
DMTF1
UROD
TOPORS
ZFP36L1
PDCL
SCML2
INTS5
AL117645
AK022162
C15orf24
FLCN
RDX
RND3
NM_080656
TAPBPL
RASSF8
RAB3GAP1
PELI2
FUSIP1
MYOCD
CTDSPL2
SLC44A1
FAM46A
SYAP1
RBM3
PRKD3
RPPH1
NOTCH2NL
NUP107
AK001099
AK001279
PER3
BNC2
STC1
MAP2K1
MED10
CACHD1
UBE2E3
CTNNA1
VASH1
CDSN
NFIA
DDR1
NOLA2
AB040883
HMGB1
SRY
IFI16
IARS
RAC3
GAPDH
COX5B
TGIF1
MED24
CDKN1A
SLC30A9
FOXN3
EGFR
NISCH
EP300
PELP1
ATF2
SMAD4
SIRT1
PGR
NCOA2
PTEN
FOXO3
CDKN2A
SMAD2
THRA
THRB
RARG
RARB
NR1I2
NR1H2
NR1H3
NR2E3
TAF11
MAP4
VDR
DAP3
SVIL
HIF1A
CDK6
CDK5
STAT3
RAF1
TP53
RELA
CAV1
MMP2
CDK3
MS4A2
HSPA8
NPAS2
MYOD1
KDR
SMYD3
APOB
MYC
NOS3
ATXN1
NUDT9
TNF
TNFRSF9
MAPK9
CDKN1B
HINFP
RIPK3
PKN1
TCEB3B
ZNF205
TRAIP
ZBTB43
DTNB
AFF4
TRIM37
