# Supplementary material for: Integrative network analysis reveals molecular mechanisms of blood pressure regulation
Source: Mol Syst Biol. 2015 Apr 16;11(4):799. doi: 10.15252/msb.20145399 (PMC4422556; doi:10.15252/msb.20145399)

Supplementary Fig S6: Screenshot of the alignment of RNA-seq reads of gene *Sh2b3* in a WT mouse and a *Sh2b3*$^{-/-}$ mouse. The RNA reads of exon 3-8 of *Sh2b3* is absent in the Sh2b3$^{-/-}$ mouse as expected. This picture is draw by Integrative Genomics Viewer (IGV) (Thorvaldsdóttir et al, 2012) .

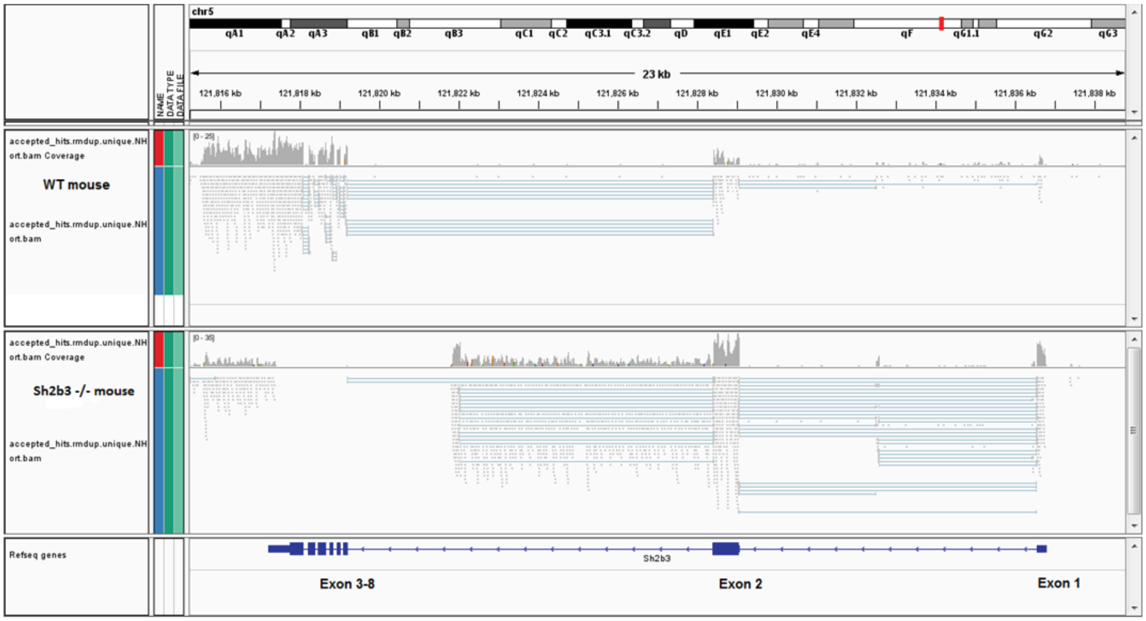

Supplement: Supplementary file 6 — Supplementary Figure S6 [file MSB-11-799-s004.pdf]
