# Supplementary material for: Integrative network analysis reveals molecular mechanisms of blood pressure regulation
Source: Mol Syst Biol. 2015 Apr 16;11(4):799. doi: 10.15252/msb.20145399 (PMC4422556; doi:10.15252/msb.20145399)

Supplementary Fig S7: A Schematic Figure of the Key Driver Analysis (KDA). In order to test if gene *G* (shown in red) is a KD or not, the subnetwork of *G* is first extracted by retrieving its 1st to 3rd-layer neighbor genes in the network. Subsequently, the enrichment of genes in a given BP gene set (shown in blue) in the subnetwork of *G* is evaluated. *G* is defined as a KD if the subnetwork of *G* is significantly enriched for genes in the tested gene set (evaluated by Fisher's Exact test; the significant threshold was Bonferroni-corrected for the number of genes in the gene network used).

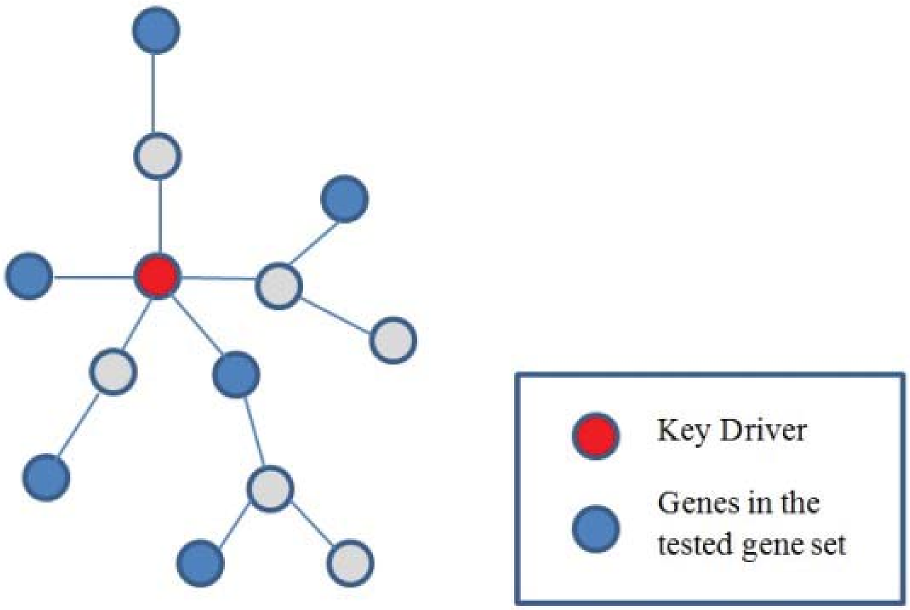

Supplement: Supplementary file 7 — Supplementary Figure S7 [file MSB-11-799-s015.pdf]
